# Tissue Nonspecific Alkaline Phosphatase (TNAP) Regulates Cranial Base Growth and Synchondrosis Maturation

**DOI:** 10.3389/fphys.2017.00161

**Published:** 2017-03-21

**Authors:** Hwa K. Nam, Monika Sharma, Jin Liu, Nan E. Hatch

**Affiliations:** Department of Orthodontics and Pediatric Dentistry, School of Dentistry, University of MichiganAnn Arbor, MI, USA

**Keywords:** bone, endochondral, chondrocyte, hypophosphatasia, craniofacial

## Abstract

Hypophosphatasia is a rare heritable disorder caused by inactivating mutations in the gene (*Alpl*) that encodes tissue nonspecific alkaline phosphatase (TNAP). Hypophosphatasia with onset in infants and children can manifest as rickets. How TNAP deficiency leads to bone hypomineralization is well explained by TNAP's primary function of pyrophosphate hydrolysis when expressed in differentiated bone forming cells. How TNAP deficiency leads to abnormalities within endochondral growth plates is not yet known. Previous studies in hypophosphatemic mice showed that phosphate promotes chondrocyte maturation and apoptosis via MAPK signaling. *Alpl*^−/−^ mice are not hypophosphatemic but TNAP activity does increase local levels of inorganic phosphate. Therefore, we hypothesize that TNAP influences endochondral bone development via MAPK. In support of this premise, here we demonstrate cranial base bone growth deficiency in *Alpl*^−/−^ mice, utilize primary rib chondrocytes to show that TNAP influences chondrocyte maturation, apoptosis, and MAPK signaling in a cell autonomous manner; and demonstrate that similar chondrocyte signaling and apoptosis abnormalities are present in the cranial base synchondroses of *Alpl*^−/−^ mice. Micro CT studies revealed diminished anterior cranial base bone and total cranial base lengths in *Alpl*^−/−^ mice, that were prevented upon injection with mineral-targeted recombinant TNAP (strensiq). Histomorphometry of the inter-sphenoidal synchondrosis (cranial base growth plate) demonstrated significant expansion of the hypertrophic chondrocyte zone in *Alpl*^−/−^ mice that was minimized upon treatment with recombinant TNAP. *Alpl*^−/−^ primary rib chondrocytes exhibited diminished chondrocyte proliferation, aberrant mRNA expression, diminished hypertrophic chondrocyte apoptosis and diminished MAPK signaling. Diminished apoptosis and VEGF expression were also seen in 15 day-old cranial base synchondroses of *Alpl*^−/−^ mice. MAPK signaling was significantly diminished in 5 day-old cranial base synchondroses of *Alpl*^−/−^ mice. Together, our data suggests that TNAP is essential for the later stages of endochondral bone development including hypertrophic chondrocyte apoptosis and VEGF mediated recruitment of blood vessels for replacement of cartilage with bone. These changes may be mediated by diminished MAPK signaling in TNAP deficient chondrocytes due to diminished local inorganic phosphate production.

## Introduction

Hypophosphatasia is a rare heritable disorder caused by inactivating mutations in the gene (*Alpl*) that encodes tissue nonspecific alkaline phosphatase (TNAP). Manifestations of hypophosphatasia in humans depend upon phenotype severity and age of onset (Rathbun, 1959; Mornet, 2007; Whyte et al., 2015). The skeletal presentation of adult onset hypophosphatasia can include osteomalacia, bone pain, bone fractures and pseudofractures (Linglart and Biosse-Duplan, 2016; Whyte, 2016). The skeletal presentation of infancy and childhood onset hypophosphatasia can include rickets, bone pain, bowed bones, bone fractures, craniosynostosis, and an abnormal craniofacial shape (Collmann et al., 2009; Linglart and Biosse-Duplan, 2016; Whyte, 2016).

*Alpl*^−/−^ mice serve as an important model of hypophosphatasia. *Alpl*^−/−^ mice develop long and craniofacial bone mineralization abnormalities similar to those seen in human infantile hypophosphatasia (Fedde et al., 1999; Liu et al., 2014). Furthermore, these mice were previously used to demonstrate that systemic treatment with a mineral-targeted recombinant form of TNAP enzyme can minimize long bone and craniofacial skeletal abnormalities when provided shortly after birth (Millán et al., 2008; Liu et al., 2015). *Alpl*^−/−^ mice are phenotypically normal at birth but quickly develop skeletal disease including rib, long bone, calvarial, and facial bone hypomineralization. Histologic evidence of chondrocyte maturation abnormalities within long bone epiphyseal and cranial base synchondroses were also reported (Narisawa et al., 1997; Fedde et al., 1999; Hessle et al., 2002; Liu et al., 2014). Together, these results indicate that TNAP is essential for both intramembranous and endochondral bone development.

Previous studies of *Alpl*^−/−^ mice also revealed important insights into mechanisms by which TNAP mediates skeletal development. Tissue nonspecific alkaline phosphatase (TNAP) is a glycosylphosphatidylinositol anchored ectoenzyme (Low and Zilversmit, 1980) that is highly expressed in differentiated bone forming cells (Millán and Whyte, 2016). TNAP activity promotes tissue mineralization by hydrolyzing inorganic pyrophosphate (PP_*i*_) to inorganic phosphate (P_*i*_) (Fedde et al., 1999), and by dephosphorylating osteopontin (Narisawa et al., 2013). PP_*i*_ and osteopontin are strong inhibitors of mineralization (Fleisch et al., 1966; Register and Wuthier, 1985; Addison et al., 2010), and crossing of *Alpl*^−/−^ mice with *Enpp1*^−/−^ and/or *Opn*^−/−^ knockout mice partially rescues the *Alpl*^−/−^ hypomineralization phenotype (Harmey et al., 2004, 2006; Anderson et al., 2005). It is therefore currently accepted that the bone hypomineralization phenotype of hypophosphatasia results largely from the accumulation of these two inhibitors, albeit in a bone site specific manner (Anderson et al., 2005).

Notably, previously reported evidence also indicates that TNAP has important functions in pre-differentiated and differentiating cells that are distinct from and in addition to its function of promoting tissue mineralization when expressed in differentiated cells. TNAP is expressed in neural progenitor cells before and after birth (Langer et al., 2007; Kermer et al., 2010), and in cranial bone rudiments days prior to the onset of matrix mineralization (Ishii et al., 2003; Sun et al., 2013). TNAP influences neural progenitor cell proliferation, differentiation and migration; and TNAP deficiency causes altered neuronal morphology and activity (Kermer et al., 2010; Sebastián-Serrano et al., 2016). TNAP also appears to directly influence the proliferation and differentiation of cranial bone progenitor cells and TNAP deficiency causes craniosynostosis (Liu et al., 2014, 2015). Of relevance to the studies reported here, TNAP is also strongly expressed in growth plate chondrocytes (Fedde et al., 1999). TNAP deficiency causes endochondral growth plate abnormalities, yet the mechanism by which TNAP deficiency alters chondrocyte maturation and the process of endochondral bone development is not yet known.

We previously showed that *Alpl*^−/−^ mice exhibit craniofacial shape abnormalities indicative of possible cranial base growth deficiency, and noted histologic abnormalities within cranial base synchondroses of juvenile *Alpl*^−/−^ mice (Liu et al., 2014). Cranial base synchondroses are bidirectional growth plates with a central resting chondrocyte zone surrounded bilaterally by proliferative and hypertrophic chondrocyte zones (Nie, 2005; Wei et al., 2016). Similar to that seen in long bones, cranial base bones elongate via an endochondral process involving chondrocyte proliferation, hypertrophy and apoptosis followed by vascular invasion and replacement of cartilage with bone. Previous studies showed that inorganic phosphate levels control chondrocyte maturation and apoptosis via MAPK signaling (Kimata et al., 2010; Miedlich et al., 2010). *Alpl*^−/−^ mice are not hypophosphatemic yet TNAP is a local generator of inorganic phosphate. Because the growth plate abnormalities of *Alpl*^−/−^ mice appear similar to those of systemically hypophosphatemic mice (Li et al., 1997; Sabbagh et al., 2005), we hypothesize that TNAP directly influences cranial base chondrocyte maturation via a similar phosphate mediated process. In support of this idea, here we demonstrate cranial base bone growth deficiency in *Alpl*^−/−^ mice, utilize primary rib chondral chondrocytes to show that TNAP influences chondrocyte maturation, apoptosis, and MAPK signaling in a cell autonomous manner; and demonstrate that similar chondrocyte signaling and maturation abnormalities are present in the cranial base synchondroses of *Alpl*^−/−^ mice.

## Materials and methods

### Animals

*Alpl*^+/−^ (TNAP^+/−^) mice were bred and genotyped to generate *Alpl*^+/+^ and *Alpl*^−/−^mice, as previously reported (Narisawa et al., 1997; Yadav et al., 2011; Liu et al., 2014). TNAP is essential for vitamin B6 metabolism (Waymire et al., 1995). Therefore, all mice were given free access to modified laboratory rodent diet 5,001 containing 325 ppm of pyridoxine to prevent seizures and improve health. All animal procedures were performed according to federal guidelines for the care and use of animals in research and/or the University of Michigan's University Committee on Use and Care of Animals.

### TNAP enzyme treatment

Recombinant TNAP protein (strensiq, previously known as asfotase-alfa) is composed of soluble human TNAP enzyme fused to the constant region of human IgG1 (Fc), and a C-terminal deca-aspartate region that confers targeting to hydroxyapatite (Nishioka et al., 2006; Whyte et al., 2012). Mice were injected subcutaneously in the subscapular region, daily, with vehicle or 8.2 mg/kg of protein starting at birth. This dose and regimen was previously shown to prevent long bone, rib, vertebral and tooth defects in murine HPP (Millán et al., 2008; McKee et al., 2011; Yadav et al., 2011; Foster et al., 2013). This protocol was also previously shown to improve cranial bone mineralization and prevent craniosynostosis in *Alpl*^−/−^mice (Liu et al., 2015).

### Scanning electron microscropy

Cranial base bones were gold coated (DESK II, Denton Vacuum) then imaged using a scanning electron microscope (JSM-7800FLV Scanning Electron Microscope, JEOL USA Inc.) with a back-scattered electron (BSE) detector and an energy-dispersive X-ray spectrometer (EDX) for quantification of carbon, calcium, and phosphate content in bone tissues using AZtecEnergy software (Oxford Instruments).

### Micro computed tomography

Whole dissected skulls were scanned at an 18 μm isotropic voxel resolution using the eXplore Locus SP micro-computed tomography imaging system (GE Healthcare Pre-Clinical Imaging, London, ON, Canada), as previously described (Liu et al., 2013, 2014). Linear distances between cranial base landmarks at the midline were generated using *Dolphin Imaging 11.0* software (Dolphin Imaging and Management Solutions, Chatsworth, CA). Linear distances included presphenoid length (distance between the most anterior aspect of the presphenoid bone and the most posterior aspect of the presphenoid bone), basisphenoid length (distance between the most anterior aspect of the basisphenoid bone and the most posterior aspect of the basisphenoid bone), basioccipitus length (distance between the most anterior aspect of the basioccipital bone and the most posterior aspect of the basioccipital bone), total cranial base length (distance between the most anterior aspect of the presphenoid bone and the most posterior aspect of the basisphenoid bone) and total skull length (distance between nasale and opisthion). No significant difference between genders was found; therefore, genders were combined for analyses (*n* = 24 per genotype and treatment group).

### Histology and histomorphometry

For qualitative histologic assessment, mouse skulls were fixed, decalcified, and embedded in paraffin. Paraffin tissue blocks were trimmed in the sagittal plane to within 4 mm of the skull midline. Eight micrometer sections parallel to the cranial base were prepared using a Leica RM2255 microtome (Leica Microsystems Inc., Buffalo Groves, IL).

For histomorphometry of cranial base synchondroses prior to the onset of craniosynostosis, 15 day-old mouse skulls were fixed, serially dehydrated, washed in isopropanol, incubated in xylene and then embedded in methyl methacrylate. Methacrylate blocks were trimmed in the sagittal plane to within 2 mm of the skull midline. Four micrometer sagittal sections of the cranial base were transferred to slides and dried overnight. Stained slides were photographed at a 10x magnification. Hypertrophic zone width and total synchondrosis widths were calculated using *NIH Image* software. Cell and synchondrosis widths were performed in triplicate and an average value for each mouse was utilized to calculate means and standard deviations per genotype and treatment group (*n* = 24 *Alpl*^−/−^ untreated mice, 8 strensiq treated *Alpl*^−/−^mice, 8 *Alpl*^+/+^ mice).

### Primary chondrocyte isolation

Because isolation of chondrocytes from murine cranial base yielded very few cells per mouse, primary rib chondrocytes were isolated from 5 day-old pups by sequential enzyme digestion to investigate a potential cell autonomous influence of TNAP on chondrocytes. Costal cartilage was isolated and digested in 2 mg/ml pronase (Sigma) at 37C for 1 h with gentle agitation. After washing with PBS, the cartilage segments were digested in 3 mg/ml collagenase D at 37C for 1 h followed by a final digestion in fresh collagenase D for 5 h. Cells were then filtered through a cell strainer and centrifuged at 400 g for 5 min. Cells were immediately counted and plated for experiments.

### Cell culture and assay

Cells were cultured in DMEM F12 media containing 10% fetal bovine serum and 1% penicillin/streptomycin. Cells were differentiated in DMEM F12 media containing 10% fetal bovine serum, 1% penicillin/streptomycin, 1x insulin/transferrin/selenium supplement, 50 ug/ml ascorbate and 5 mM NaPO_4_. Alkaline phosphatase enzyme activity was assayed using the colorimetric substrate, NBT/ BCIP (Sigma). For Alcian Blue staining, cells were washed with phosphate buffered saline and fixed with 10% formalin. The cells were then stained with a 1% Alcian Blue solution (1% alcian blue in 60% ethanol/40% acetic acid). After destaining in 60% ethanol/40% acetic acid, cells were photographed and wells were scanned. Scans were quantified by densitometry using *NIH Image* software. To assay cellular proliferation, cells were seeded and grown DMEM F12 media containing 10% fetal bovine serum and 1% penicillin/streptomycin. Cells were plated at equivalent numbers, grown under equivalent conditions, monolayer cells were washed to removed dead cells, cells were trypsinized then stained with trypan blue, and then counted in triplicate for each time point using a hemocytometer. To assay cellular apoptosis, a Cell Death Detection kit (Roche) was utilized according to the manufacturer's instructions. This assay uses antibodies directed against DNA and histones, to quantify mono- and oligonucleosomes that are released into the cytoplasm of cells that die from apoptosis. Briefly, 10,000 cells were seeded into 96-well plates and grown in media containing 10 or 0.5% FBS for 48 h. Cell lysate was utilized to quantify apoptosis by a colorimetric reaction and absorbance was measured at 405 nm (reference wavelength of 490 nm). For RNA analysis, RNA was isolated using Trizol reagent (Invitrogen) following manufacturer protocols. mRNA levels were assayed by reverse transcription and real time PCR. Real time PCR was performed utilizing the murine bActin primer/probe set 4352933E, the Sox9 primer/probe set Mm00448840_m1, the Col2a1 primer/probe set Mm01309565_m1, the Col10a1 primer/probe set Mm00487041_m1, the Runx2 primer/probe set Mm00501578_m1, the Alpl primer/probe set Mm00475834_m1, the VEGF primer/probe set Mm00437306_m1, and Taqman Universal PCR Master Mix (Applied Biosystems). Real-time PCR was performed on a ViiA7 thermocyler (Life Technologies) and quantified by comparison to a standard curve.

### Immunoblotting

Preparation of cell lysate was achieved by solubilization in RIPA buffer (50 mM Tris-Cl pH 7.4, 150 mM NaCl, 1% NaDeoxycholate, 1% Triton-X 100, 0.1% SDS) containing 1x protease inhibitor cocktail (Sigma), followed by removal of insoluble material by centrifugation at 12,000 rpm for 10 min. Prior to loading, 5x Laemmli loading buffer was added to a final 1x concentration, samples were boiled for 3 min, and then iced. Samples were separated by SDS polyacrylamide gel electrophoresis and transferred onto Immobilon (Millipore). Immunoreactive protein bands were visualized by incubation with MAPK and phospho-MAPK antibodies (Cell Signaling), HRP conjugated secondary antibody (Abcam) and enhanced chemiluminescence (Pierce).

### Immunofluorescence

Skulls of 15 day-old mice were fixed and paraffin embedded. Fresh 7 μm sagittal setions of the cranial base were immunostained using Annexin V antibody (Abcam), anti VEGF antibody (Abcam), or cleaved caspase 3 antibody (Cell Signaling). After primary antibody incubation, sections were incubated with Alexafluor 555 goat anti-rabbit IgG (Molecular Probes), then mounted in ProLong Gold antifade reagent with DAPI (Invitrogen). Images were captured using Nikon Eclipse E800 microscope. Image J software was used for immunofluorescent stain quantification to calculate means and standard deviations per genotype (*n* = 3 mice per genotype).

### Immunohistochemistry

Skulls of 5 day-old mice were fixed and paraffin embedded. Fresh 8 μm sagittal sections of the cranial base were immunostained using MAPK, and phosphorylated-MAPK primary antibodies (Cell Signaling), HRP-conjugated anti-rabbit secondary antibody (Abcam), an antigen unmasking solution (Vector Labs) and 3,3'-diaminobenzidine (DAB) colorimetric detection (Abcam). The number of brown stained cells and the number of total cells present within the synchondroses were counted. Three sections per mouse were calculated and an average value per mouse was utilized to calculate means and standard deviations per genotype (*n* = 3 mice per genotype).

### Statistical analysis

All quantified data are presented as means ^+/−^ standard deviations. For quantifiable data, statistical significance between genotypes and treatment groups was established using the student's *t*-test. A *p* < 0.05 is considered as significant.

## Results

### TNAP deficiency causes cranial base abnormalities in *Alpl*^−/−^ mice

Similar to that which we previously reported (Liu et al., 2014), H&E staining of the cranial base demonstrated gross abnormalities of both the sphenooccipital (SOS) and intersphenoidal (ISS) synchondroses in *Alpl*^−/−^ mice (Supplementary Figure 1). Both growth plates appeared widened, and conspicuous expansion of hypertrophic chondrocyte zones was evident. Also of note were the excess cortical and trabecular bone matrix deposition, and loss of marrow space in *Alpl*^−/−^ cranial base bones.

**Figure 1:**
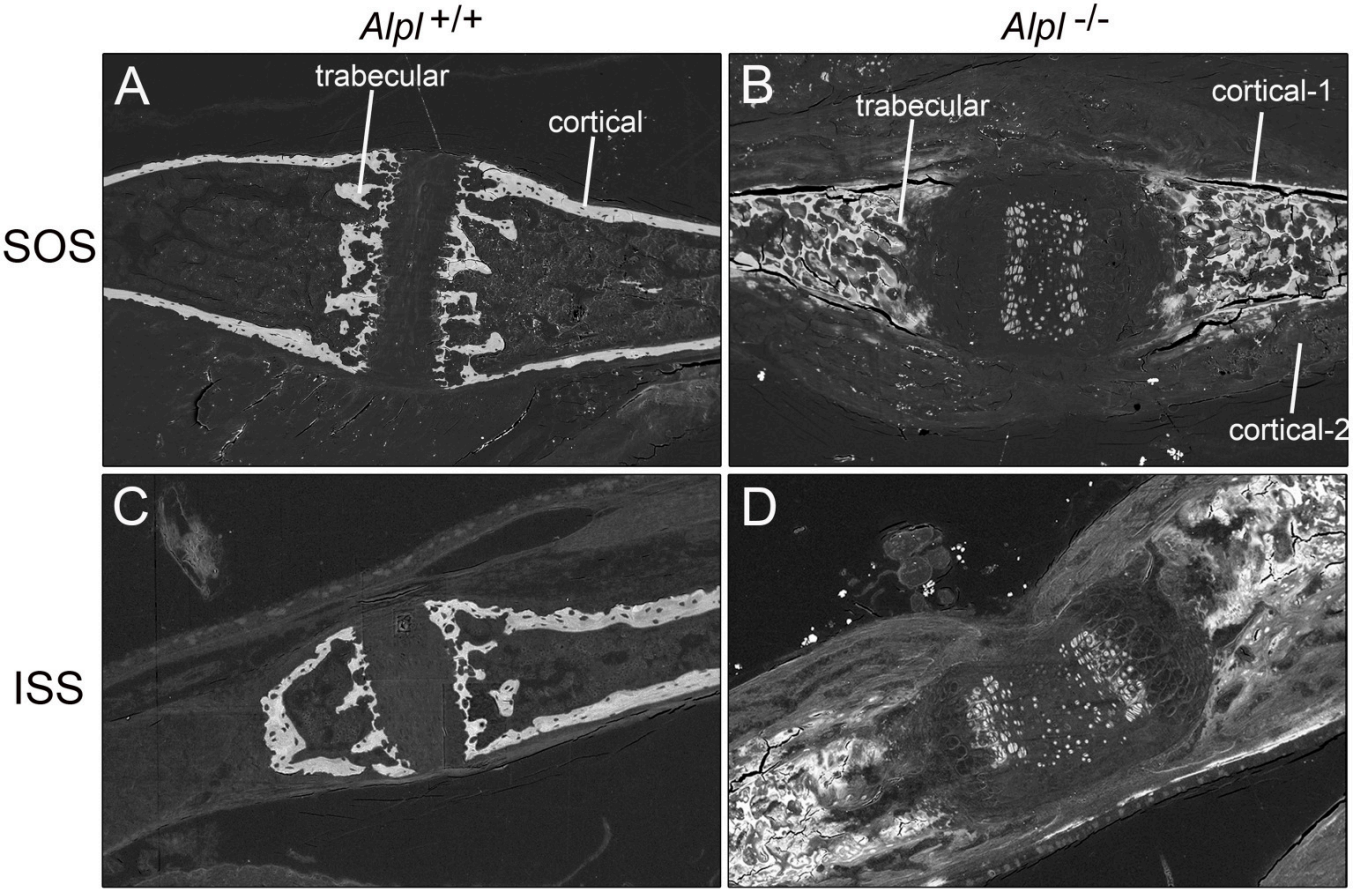
**Backscattered electron detection reveals mineralization differences in the cranial base of 20 day-old ***Alpl***^**−/−**^ and ***Alpl***^**+/+**^ mice**. Cranial base bones surrounding the sphenoccipital **(A,B)** and intersphenoid **(C,D)** synchondroses are shown. Mineral is present within the presumed marrow space and within the cartilaginous growth plates of cranial bones isolated from *Alpl*^−/−^ **(B,C)** but not *Alpl*^+/+^ **(A,B)** mice. Cortical bone matrix that is farther away from the presumed marrow space (cortical-2) appears less mineralized than cortical bone matrix that is closer to the presumed marrow space (cortical-1) in *Alpl*^−/−^ mice.

Scanning electron microscopy (SEM) with backscattered electron detection (BSE) of cranial base bones from 20 day-old mice revealed mineralization within both the ISS and SOS growth plates, and within the presumed marrow space of *Alpl*^−/−^ mice (Figure 1). In cranial base trabecular bone surrounding both the ISS and SOS, energy dispersive X-ray spectroscopy (EDX) demonstrated increased calcium and phosphate content in *Alpl*^−/−^ mice, as compared to that seen in cranial base bones of wild type mice (Table 1). Calcium/phosphate ratios were similar in *Alpl*^−/−^ and *Alpl*^+/+^ mice, indicating that the higher mineral content seen in the trabecular bone of *Alpl*^−/−^ mice was likely apatitic. *Alpl*^−/−^ cortical bone located immediately adjacent to the presumed marrow cavity had similar calcium and phosphate contents plus a similar calcium/phosphate ratio to that of *Alpl*^+/+^ cortical bone. In contrast, *Alpl*^−/−^ cortical bone located farther away from the presumed marrow space had diminished calcium and phosphate content, and a similar calcium/phosphate ratio to that of *Alpl*^+/+^ cortical bone. *Alpl*^−/−^ cortical bone was assessed in two regions while *Alpl*^+/+^ cortical bone was assessed in only one region, because cortical bone thickening was only present in cranial base cortical bone of *Alpl*^−/−^ mice.

**Table 1:** **Energy dispersive X-ray Spectroscopy**.

**Genotype**	**Region**	**Bone**	**Carbon (percent by weight)**	**Calcium (percent by weight)**	**Phosphate (percent by weight)**	**Calcium/ phosphate ratio**
*Alpl*^+/+^	SOS	Trabecular	28.7 ± 2.6	48.6 ± 1.9	23.3 ± 0.8	2.1 ± 0.0
*Alpl*^−/−^	SOS	Trabecular	22.3 ± 1.1^a^	52.6 ± 1.0^a^	25.1 ± 0.6^a^	2.1 ± 0.1
*Alpl*^+/+^	ISS	Trabecular	30.8 ± 5.3	46.9 ± 3.6	22.3 ± 1.7	2.1 ± 0.0
*Alpl*^−/−^	ISS	Trabecular	18.7 ± 2.6^a^	55.4 ± 1.9^a^	25.9 ± 0.8^a^	2.1 ± 0.0
*Alpl*^+/+^	SOS	Cortical	31.1 ± 3.9	46.6 ± 2.5	22.3 ± 1.5	2.1 ± 0.0
*Alpl*^−/−^	SOS	Cortical–1	44.6 ± 9.4^a^	38.5 ± 6.3^a^	16.9 ± 3.3^a^	2.3 ± 0.2
*Alpl*^−/−^	SOS	Cortical–2	56.8 ± 5.8^a^	29.4 ± 3.6^a^	13.9 ± 2.2^a^	2.1 ± 0.1
*Alpl*^+/+^	ISS	Cortical	33.2 ± 4.1	45.5 ± 2.7	21.2 ± 1.4	2.2 ± 0.0
*Alpl*^−/−^	ISS	Cortical–1	33.0 ± 12.0	46.1 ± 7.9	20.1 ± 4.1	2.2 ± 0.1
*Alpl*^−/−^	ISS	Cortical–2	51.2 ± 2.5^a^	32.9 ± 1.4^a^	15.8 ± 1.1^a^	2.1 ± 0.1

a *Indicates statistical significance between genotypes*.

### Recombinant TNAP enzyme therapy rescues cranial base defects in Alpl^−/−^ mice

**Figure 2:**
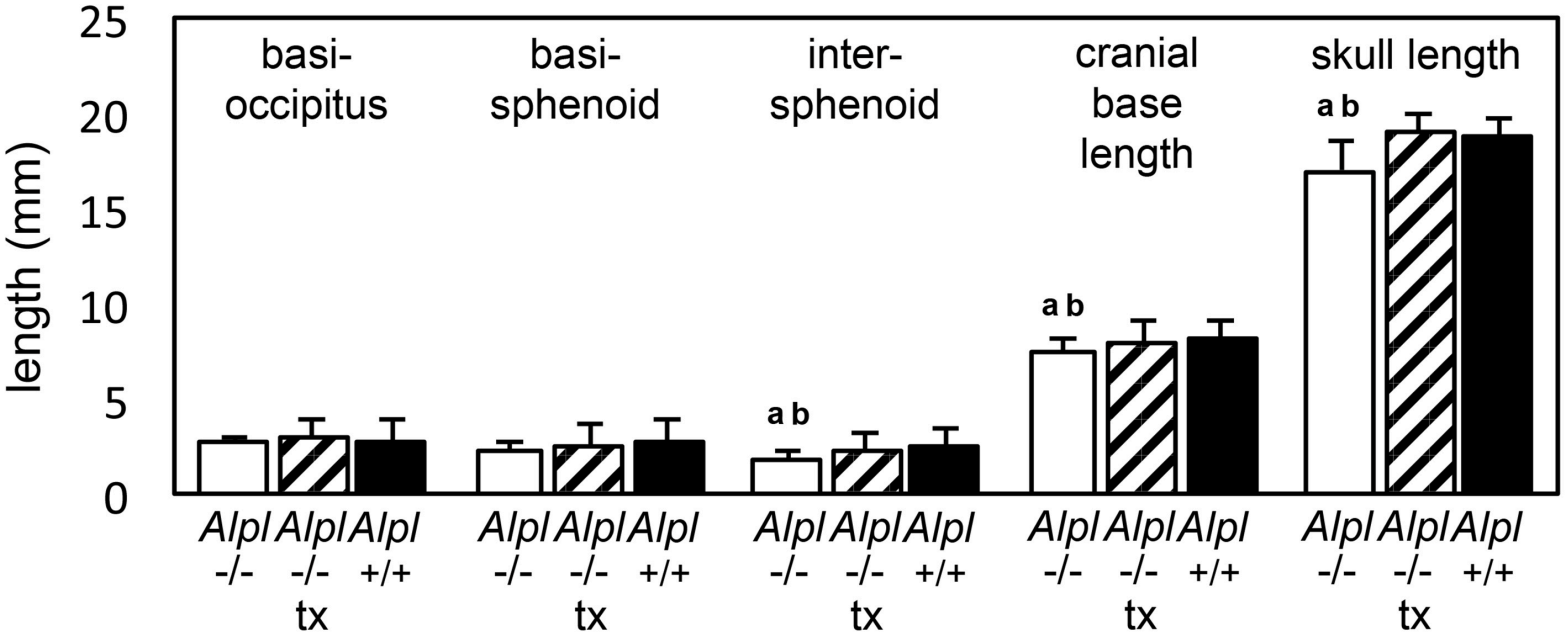
**Rescue of cranial base bone lengths by treatment with mineral-targeted recombinant TNAP enzyme in ***Alpl***^**−/−**^ mice**. Anterior-posterior bone lengths of cranial base bones taken at the midline from micro CT scans of 15 day-old untreated *Alpl*^−/−^ (white bars), 8.2 mg/kg/day treated *Alpl*^−/−^ (striped bars), and *Alpl*^+/+^ (black bars) mouse skulls are shown. Length of the intersphenoid bone, total cranial base length and total skull length are diminished in untreated *Alpl*^−/−^mice when compared to treated *Alpl*^−/−^ or *Alpl*^+/+^ mice. ^a^*p* < 0.05 significant difference between genotypes. ^b^*p* < 0.05 significant difference between treatment groups.

We previously showed that post-natal treatment with a mineral targeted recombinant form of TNAP enzyme (Strensiq) prevents craniofacial skeletal shape abnormalities in these mice (Liu et al., 2015). To determine if recombinant TNAP enzyme replacement therapy influences the cranial base in *Alpl*^−/−^ mice, here we quantified changes in cranial base bone lengths, to verify consistency of cranial base growth abnormalities and efficacy of TNAP enzyme replacement for rescuing cranial base bones in *Alpl*^−/−^ mice. Results showed that while basioccipital cranial base bones were not different between genotypes, the basisphenoid and presphenoid cranial base bones were significantly shorter in vehicle treated *Alpl*^−/−^ mice as compared to *Alpl*^+/+^ mice (Figure 2). Total length of the cranial base and total skull length were also significantly shorter in vehicle treated *Alpl*^−/−^ mice as compared to *Alpl*^+/+^ mice. Treatment with 8.2 mg/kg/day of recombinant TNAP enzyme increased apparent length of the basisphenoid and presphenoid bones, although not to the extent of that seen in *Alpl*^+/+^ mice. Importantly, treatment with mineral-targeted TNAP enzyme increased total cranial base length similar to that seen in *Alpl*^+/+^ mice. As previously reported, total skull length was also rescued upon treatment with recombinant TNAP (Liu et al., 2015). These results demonstrate that TNAP mediates anterior cranial base bone growth.

**Figure 3:**
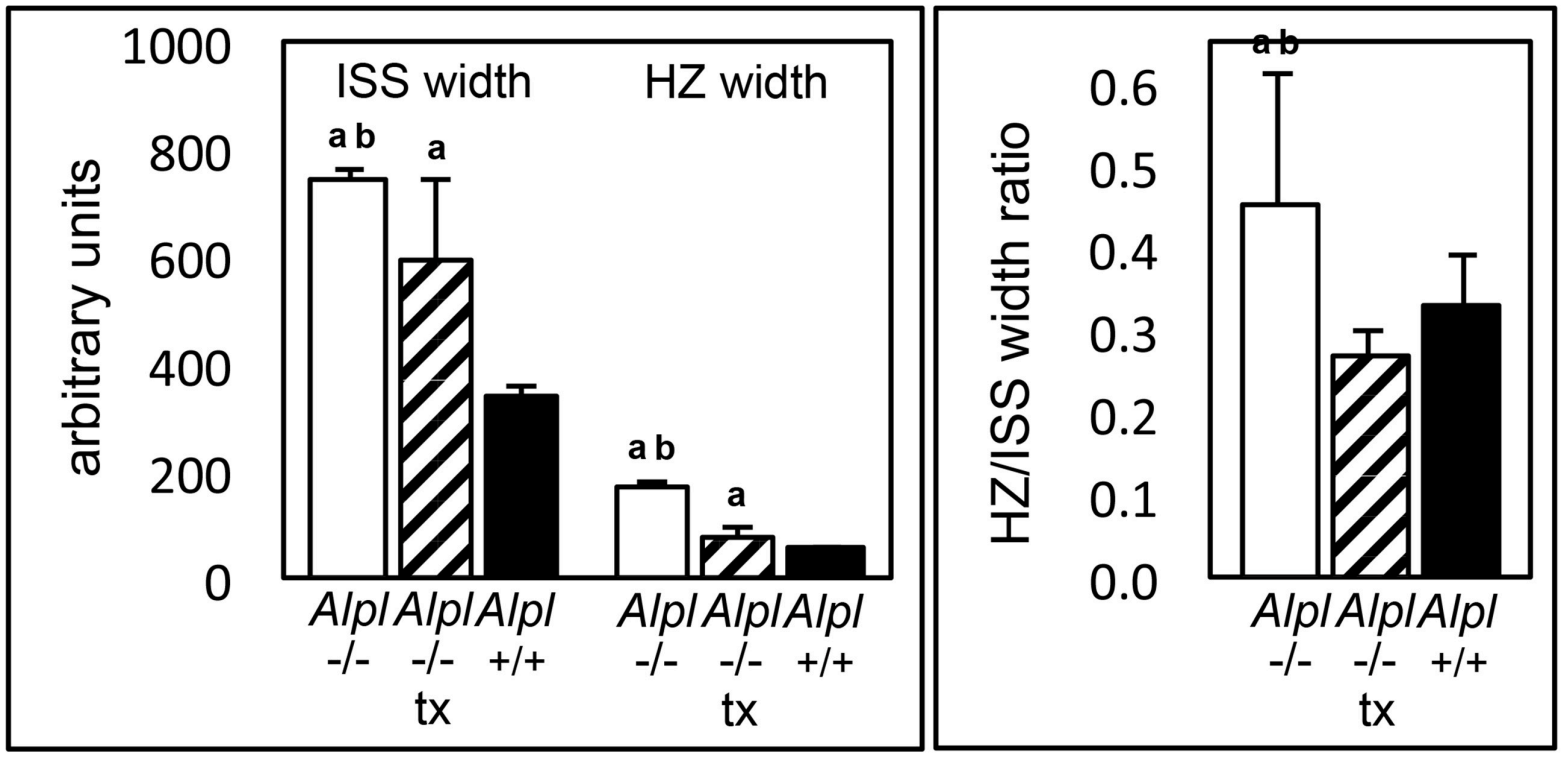
**Rescue of hypertrophic zone expansion by treatment with mineral-targeted recombinant TNAP enzyme in ***Alpl***^**−/−**^ mice**. ISS hypertrophic zone widths and total ISS widths of 15 day-old untreated *Alpl*^−/−^ (white bars), 8.2 mg/kg/day treated *Alpl*^−/−^ (striped bars), and *Alpl*^+/+^ (black bars) were measured. The hypertrophic zone width and hypertrophic zone width per total ISS width are increased in untreated *Alpl*^−/−^mice, when compared to treated *Alpl*^−/−^ or *Alpl*^+/+^ mice. ^a^*p* < 0.05 significant difference between genotypes. ^b^*p* < 0.05 significant difference between treatment groups.

Because micro CT analysis only allows for quantification of mineralized tissues, we also performed histomorphometry on cranial base growth plates. We focused on analysis of the inter-sphenoidal synchondrosis because the micro CT based assessment of cranial base bone lengths indicated that anterior cranial base bones were more affected by genotype and treatment than posterior cranial base bones. Results showed that the intersphenoidal synchondrosis was wider in untreated *Alpl*^−/−^ mice by approximately 2 weeks after birth, and that treatment with recombinant TNAP enzyme partially normalized this phenotype (Figure 3). Width of the hypertrophic chondrocyte zone was on average more than 3x greater in untreated *Alpl*^−/−^ mice when compared to *Alpl*^+/+^ mice. Treatment significantly decreased width of the hypertrophic zone in *Alpl*^−/−^ mice, although this zone was still expanded compared to that seen in *Alpl*^+/+^ mice. Finally, the ratio of hypertrophic zone width to total width of the intersphenoidal synchondrosis was larger in untreated *Alpl*^−/−^ mice compared to *Alpl*^+/+^ mice. In contrast, the ratio of hypertrophic zone width to total width of the intersphenoidal synchondrosis was not larger in treated *Alpl*^−/−^ mice, when compared to *Alpl*^+/+^ mice. These results demonstrate that TNAP mediates cranial base growth plate maturation and that post-natal treatment with mineral-targeted TNAP enzyme is efficacious for minimizing cranial base growth plate abnormalities in *Alpl*^−/−^ mice.

### TNAP deficiency directly influences primary chondrocyte behavior

**Figure 4:**
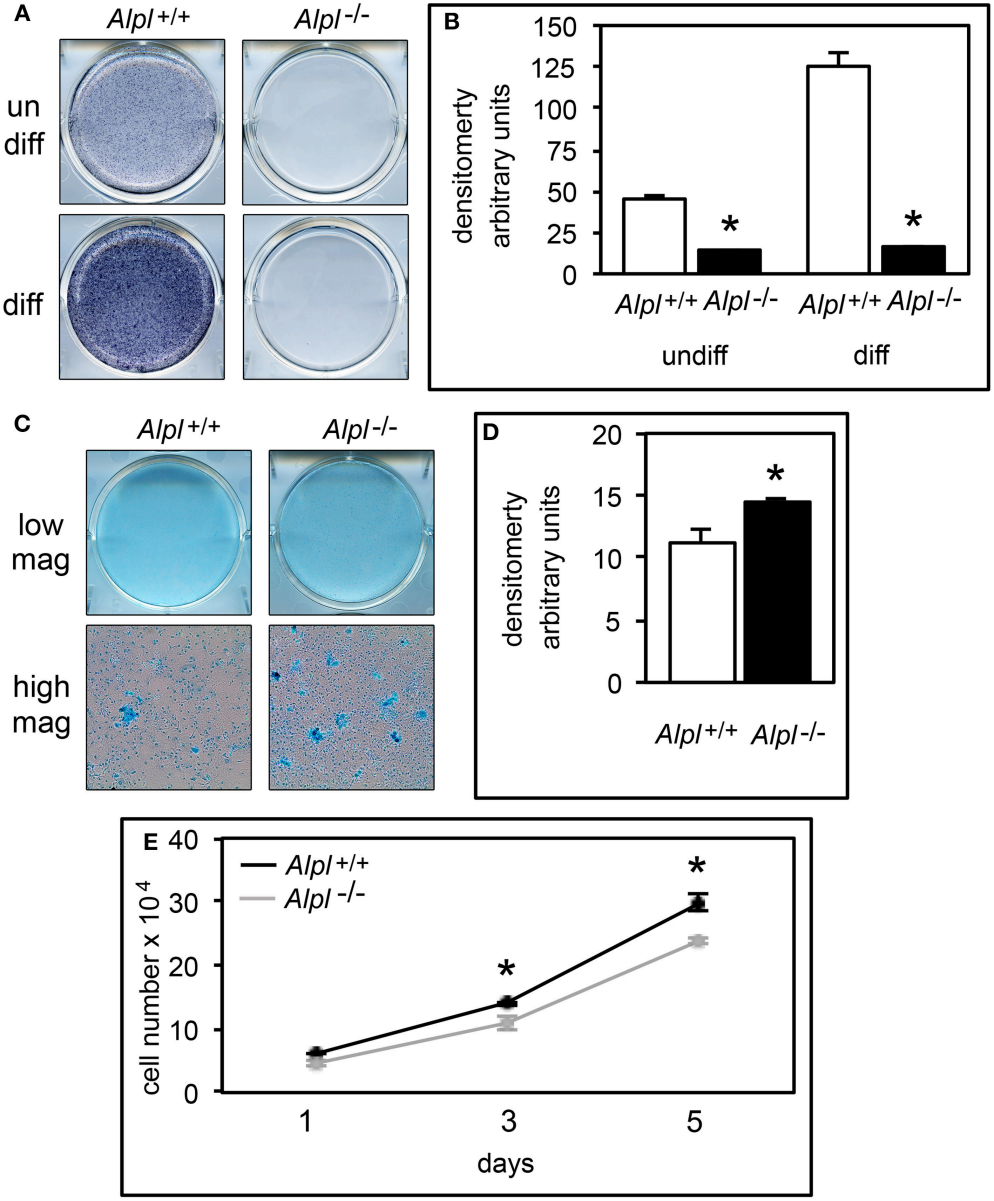
***Alpl***^**−/−**^ **primary chondrocytes exhibit increased proteoglycan accumulation and diminished proliferation. (A,B)** Primary rib chondrocytes isolated from *Alpl*^+/+^ and *Alpl*^−/−^ mice were cultured with or without ascorbate to induce chondrocyte differentiation. TNAP enzyme activity was visualized by incubation of cells with a colorimetric substrate and quantified by densitometry. **(C,D)** Primary chondrocytes were cultured under chondrocyte differentiation conditions for 15 days then stained with Alcian Blue. Staining was quantified by densitometry. **(E)** Cells were stained with trypan blue and counted at indicated time points after plating to assay for proliferation (black line = *Alpl*^+/+^; gray line = *Alpl*^−/−^). ^*^p < 0.05 between genotypes.

To initiate an investigation into mechanisms by which TNAP deficiency causes growth plate abnormalities and determine if TNAP influences chondrocytes in a cell autonomous manner, we next isolated primary rib chondral chondrocytes from *Alpl*^−/−^ and *Alpl*^+/+^ mice. Alkaline phosphatase staining confirmed significantly diminished alkaline phosphatase activity in chondrocytes isolated from *Alpl*^−/−^ mice (Figures 4A,B). Accumulation of proteoglycans as assessed by staining with Alcian Blue was increased in *Alpl*^−/−^ chondrocytes, when compared to *Alpl*^+/+^ chondrocytes (Figures 4C,D). Proliferation of chondrocytes isolated from *Alpl*^−/−^ mice was diminished when compared to cells isolated from *Alpl*^+/+^ mice (Figure 4E).

**Figure 5:**
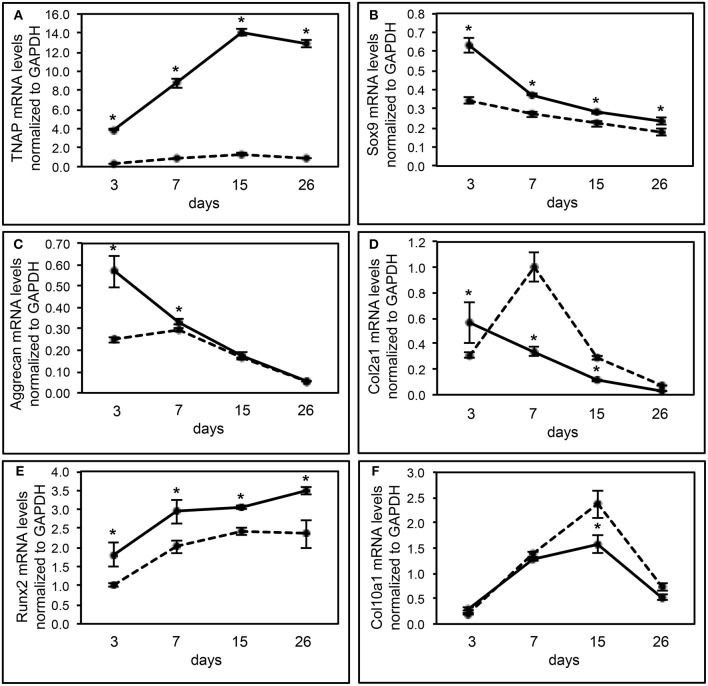
***Alpl***^**−/−**^ **primary chondrocytes exhibit abnormal gene expression during differentiation. (A–F)** Primary rib chondrocytes isolated from *Alpl*^+/+^ and *Alpl*^−/−^ mice were cultured to induce chondrocyte differentiation. Experiments were performed in triplicate. RNA was isolated at indicated time points. TNAP, sox9, aggrecan, Col2a1, Runx2, and Col10a1 mRNA levels were measured by real time PCR. Results are presented as normalized to β-Actin. Solid lines = *Alpl*^+/+^ cells, dashed lines = *Alpl*^−/−^ cells. ^*^p < 0.05 between genotypes.

We also performed real time PCR analysis of gene expression of *Alpl*^−/−^ and *Alpl*^+/+^ chondrocytes undergoing differentiation *in vitro*, to further investigate the influence of TNAP on chondrocyte differentiation (Figure 5). As expected, results showed minimal TNAP mRNA expression at all time points in cells isolated from *Alpl*^−/−^ mice. Sox9 mRNA expression levels were high early and then decreased at later time points of chondrocyte differentiation in *Alpl*^+/+^ primary chondrocytes. Sox9 mRNA levels were significantly lower in *Alpl*^−/−^ as compared to *Alpl*^+/+^ cells at all time points. Aggrecan mRNA expression levels were high early and then decreased at later time points of chondrocyte differentiation in *Alpl*^+/+^ primary chondrocytes. Aggrecan mRNA levels were significantly lower in *Alpl*^−/−^ as compared to *Alpl*^+/+^ cells at days 3 and 7 of differentiation. Col2a1 mRNA expression levels were high early and then decreased at later time points of chondrocyte differentiation in *Alpl*^+/+^ primary chondrocytes. Col2a1 mRNA levels were significantly lower at day 3 and significantly higher at days 7 and 15 in *Alpl*^−/−^ cells, as compared to *Alpl*^+/+^ cells. Runx2 mRNA mRNA expression levels increased over the time course of differentiation in *Alpl*^+/+^ primary chondrocytes. Runx2 mRNA levels were significantly lower in *Alpl*^−/−^ as compared to *Alpl*^+/+^ cells at all time points. Col10a1 mRNA expression levels peaked at day 15 of differentiation in *Alpl*^+/+^ primary chondrocytes. Col10a1 mRNA levels were significantly higher in *Alpl*^−/−^ as compared to *Alpl*^+/+^ cells at day 15.

### TNAP deficiency diminishes chondrocyte apoptosis

**Figure 6:**
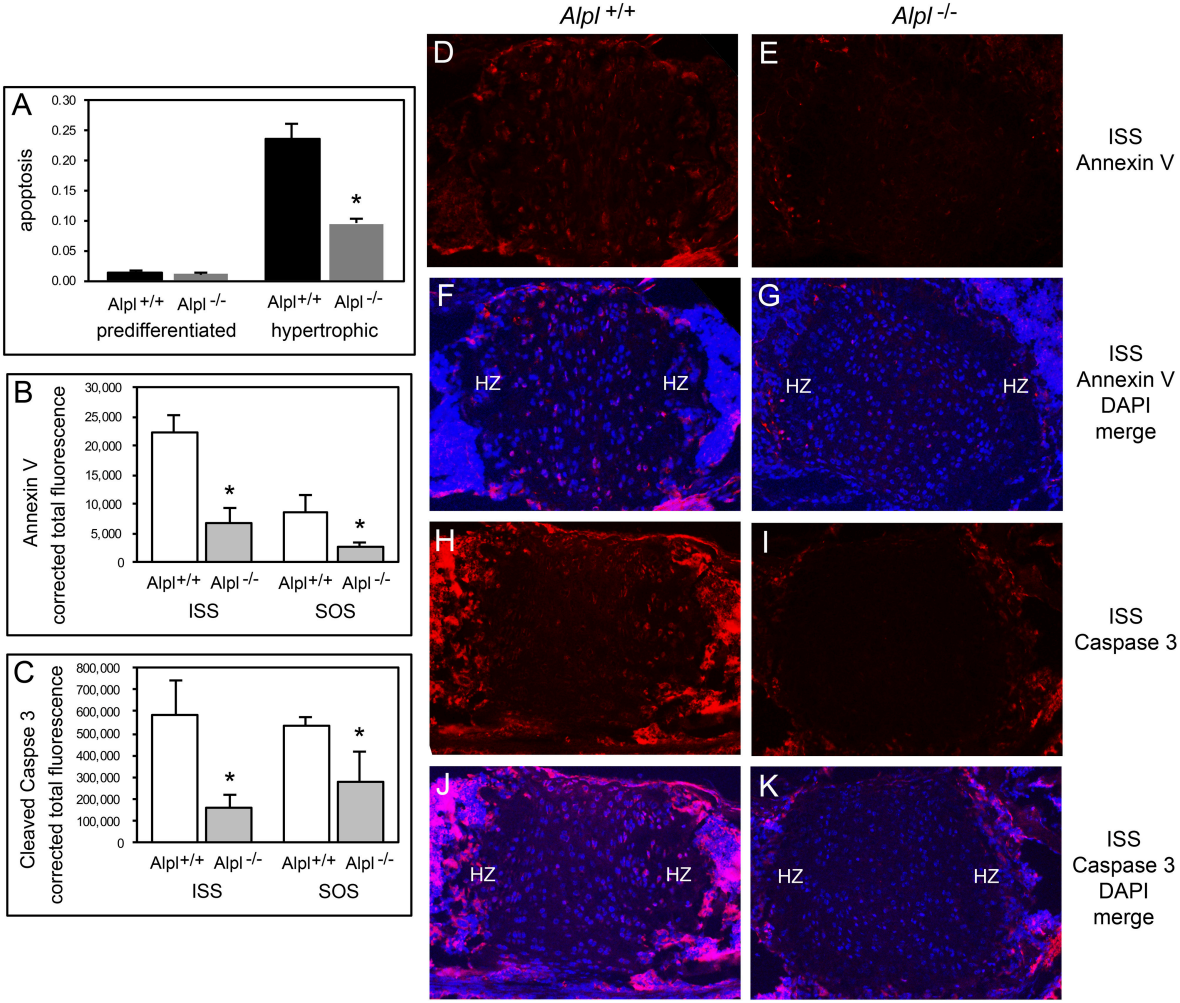
**Diminished apoptosis of ***Alpl***^**−/−**^ hypertrophic chondrocytes. (A)** Undifferentiated rib chondrocytes and differentiated rib hypertrophic chondrocytes from *Alpl*^+/+^ and *Alpl*^−/−^ mice were cultured in media containing 10% FBS. Generation of apoptotic changes in DNA was assayed by a colorimetric reaction. Results show no difference in the tendency for pre-differentiated cells to undergo apoptosis and a significantly diminished tendency for differentiated hypertrophic chondrocytes to undergo apoptosis. **(B-K)** ISS and SOS cranial base tissues were stained for Annexin V and cleaved Caspase 3 markers of apoptosis by immunofluorescence. Images show diminished expression of Annexin V in *Alpl*^−/−^ **(E,G)** compared to *Alpl*^+/+^ **(D,F)** ISS, and diminished expression of cleaved Caspase 3 in *Alpl*^−/−^ **(I,K)** compared to *Alpl*^+/+^ **(H,J)** ISS. Quantification of immunofluorescent stains within the synchondroses from sections of three mice per genotype demonstrates significant differences in Annexin V **(B)** and cleaved Caspase 3 **(C)** expression in the ISS and SOS of *Alpl*^−/−^ when compared to *Alpl*^+/^mice. ^*^p < 0.05 between genotypes. (HZ hypertrophic chondrocyte zone).

Hypertrophic chondrocyte apoptosis is an essential component of endochondral bone growth, and diminished apoptosis could explain the hypertrophic zone expansion seen in *Alpl*^−/−^ mice. We performed *in vitro* assays on rib chondral chondrocytes and *in vivo* assays on cranial base tissues to determine if apoptosis was diminished in the synchodroses of *Alpl*^−/−^ mice. *In vitro* assays revealed no significant differences in apoptosis of chondrocytes isolated from *Alpl*^−/−^ mice as compared to *Alpl*^+/+^ mice before differentiation, but significantly decreased apoptosis of chondrocytes isolated from *Alpl*^−/−^ mice after differentiation into hypertrophic chondrocytes (Figure 6A). Immunofluorescent staining for Annexin V and caspase 3 markers of apoptosis also revealed significantly diminished apoptosis of growth plate chondrocytes in both the ISS and SOS cranial base synchondroses of 15 day old *Alpl*^−/−^ mice (Figures 6B–K).

### TNAP deficiency diminishes chondrocyte VEGF expression

**Figure 7:**
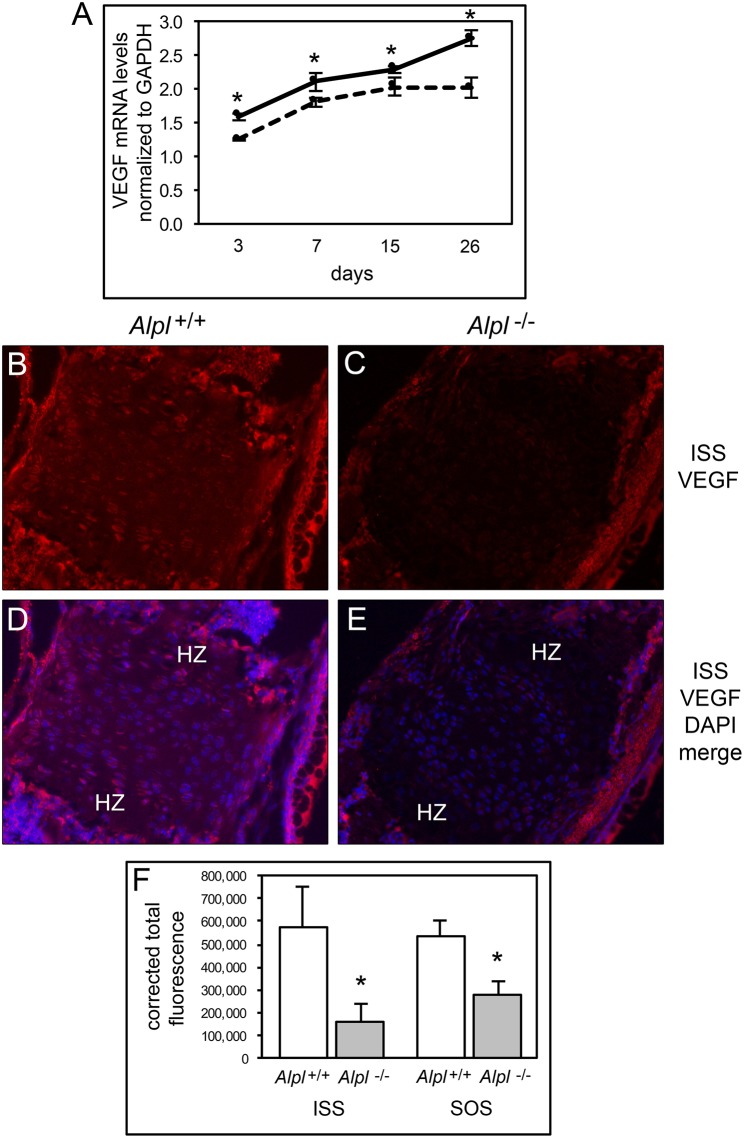
**Diminished VEGF expression in ***Alpl***^**−/−**^ chondrocytes. (A)** Primary rib chondrocytes isolated from *Alpl*^+/+^ and *Alpl*^−/−^ mice were cultured to induce chondrocyte differentiation and RNA was isolated at indicated time points. VEGF mRNA levels were measured by real time PCR. Results are presented as normalized to β-Actin. Solid lines = *Alpl*^+/+^ cells, dashed lines = *Alpl*^−/−^ cells. ^*^p < 0.05 between genotypes. **(B-F)** ISS and SOS cranial base tissues were stained for VEGF by immunofluorescence. Images show diminished VEGF expression in the ISS of *Alpl*^−/−^ **(C,E)** as compared to *Alpl*^+/+^ **(B,D)** mice. **(F)** Quantification of immunofluorescent stain within the synchondroses from sections of three mice per genotype demonstrates significant differences in VEGF expression in the ISS and SOS of *Alpl*^−/−^ when compared to *Alpl*^+/^mice. ^*^p < 0.05 between genotypes. (HZ hypertrophic chondrocyte zone).

During endochondral bone growth, hypertrophic chondrocytes secrete VEGF which stimulates vascular ingrowth ultimately leading to replacement of cartilage with bone, and inhibition of VEGF signaling causes hypertrophic chondrocyte zone expansion with diminished bone growth (Gerber et al., 1999; Carlevaro et al., 2000). Therefore, we next sought to determine if VEGF expression was diminished in cells and tissues of *Alpl*^−/−^ mice. VEGF mRNA levels were significantly lower in *Alpl*^−/−^ as compared to *Alpl*^+/+^ rib primary chondrocytes at all time points, and appeared to plateau in *Alpl*^−/−^ cells, as oppose to the increase in VEGF expression seen in in *Alpl*^+/+^ cells at later differentiation time points (Figure 7A). VEGF expression was also significantly diminished within the hypertrophic chondrocyte zones of *Alpl*^−/−^ cranial base synchondroses at 15 days after birth (Figures 7B–F).

### MAPK activity is diminished in cranial base synchondroses of *Alpl*^−/−^ mice

**Figure 8:**
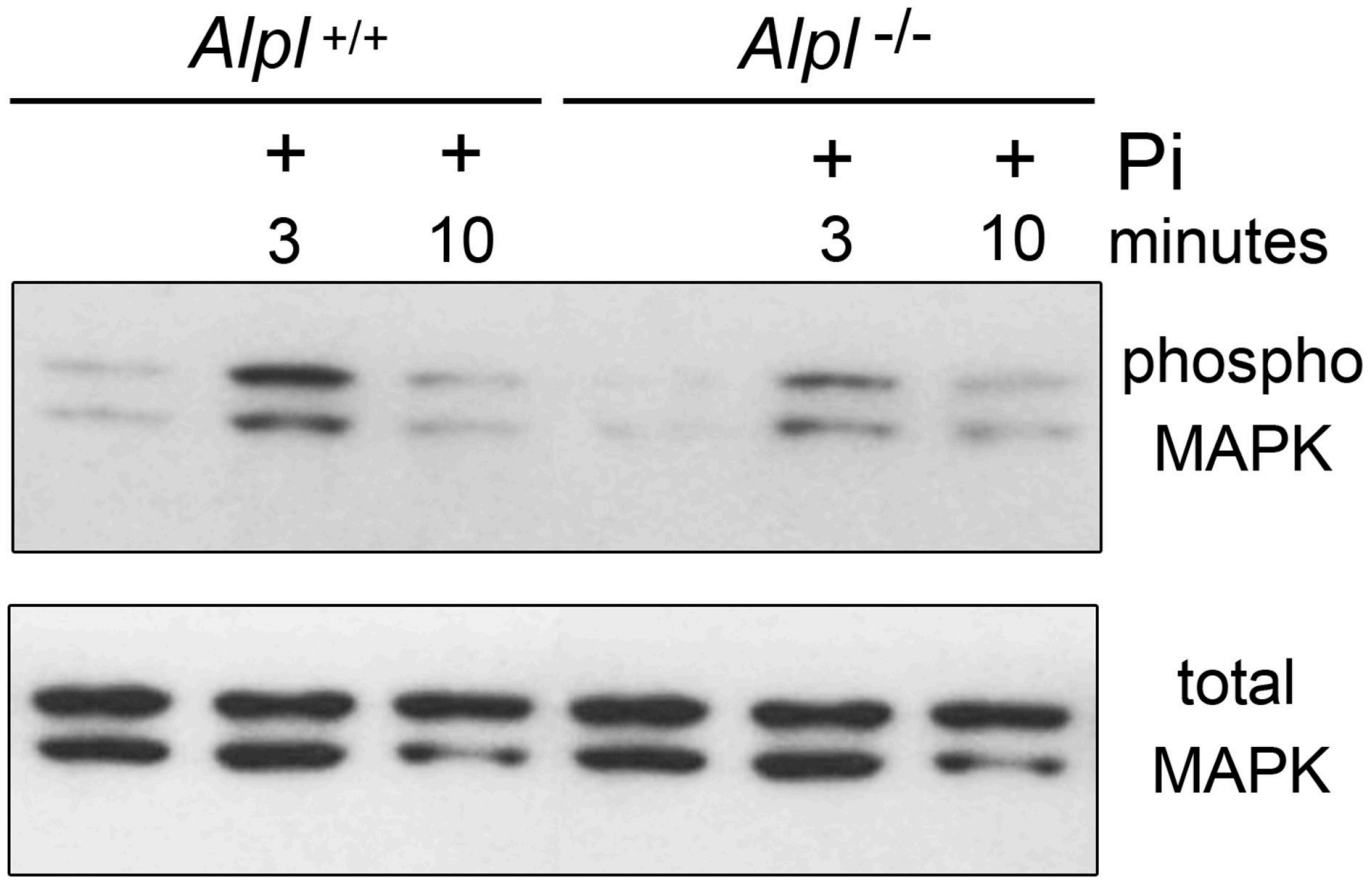
**MAPK signaling is diminished in ***Alpl***^**−/−**^ chondrocytes**. Cell lysate was isolated from primary rib chondrocytes of *Alpl*^−/−^ and *Alpl*^+/+^ mice after culture with/without inorganic phosphate. MAPK activity was assessed by immunoblotting for phosphorylated and total MAPK protein. Results show diminished basal and phosphate induced levels of phosphorylated MAPK protein in lysate of *Alpl*^−/−^ cells.

**Figure 9:**
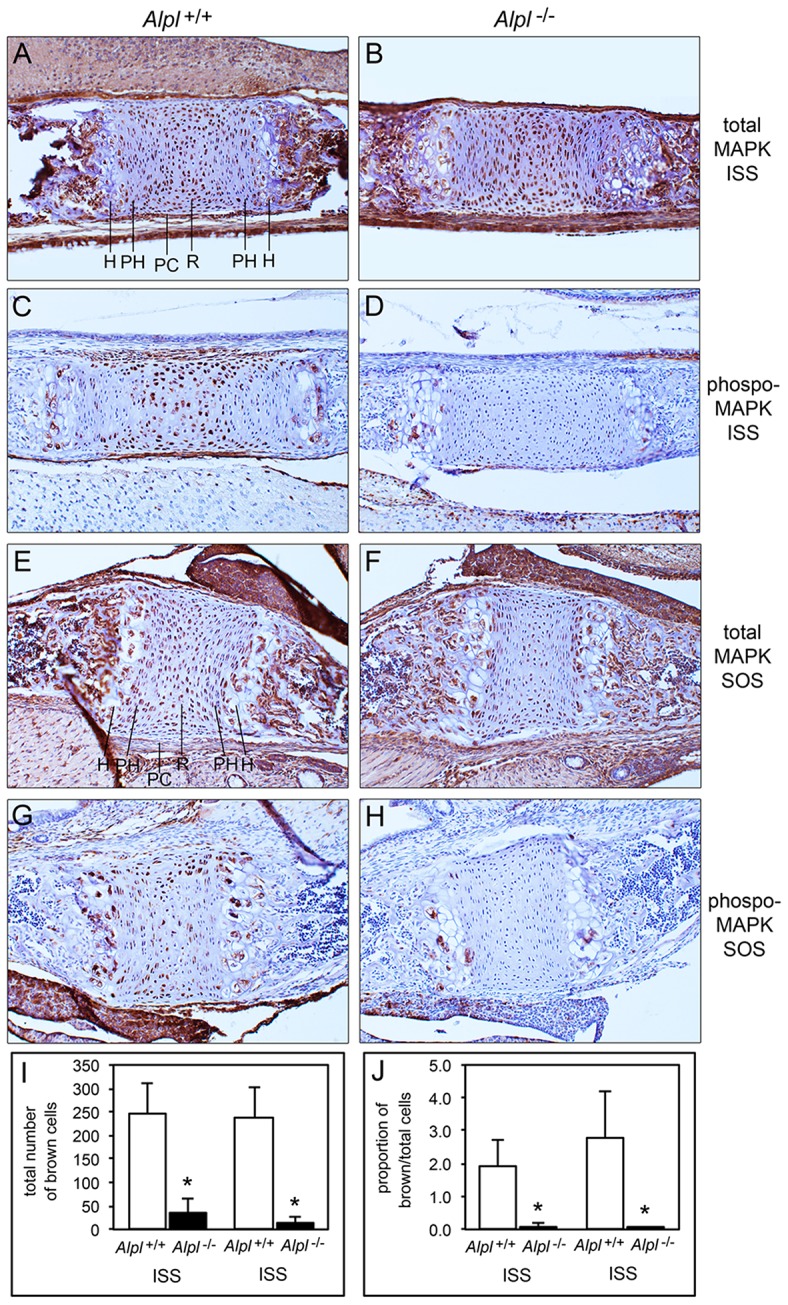
**MAPK signaling is diminished in ***Alpl***^**−/−**^ cranial base synchondroses**. Sagittal sections of cranial base synchondroses from 5 day-old mice were stained for total MAPK and phosphorylated MAPK protein. Total MAPK protein levels appear similar in the SOS and ISS of *Alpl*^+/+^ **(A,E)** and *Alpl*^−/−^ **(B,F)** mice. Phosphoryated MAPK protein levels appear diminished in the SOS and ISS of *Alpl*^−/−^ **(D,H)**, as compared to *Alpl*^+/+^ **(C,G)** mice. Stains were quantified by brown stained cell counting, and normalized to the total cell count. **(I,J)** Quantification of results from three mice per genotype demonstrates consistent and significantly diminished levels of phosphoryated MAPK in both the SOS and ISS of *Alpl*^−/−^ mice. ^*^p < 0.05 between genotypes. H, hypertrophic zone; PH, prehypertrophic zone; PC, perichondrium; R, resting zone.

Because diminished MAPK signaling mediates the hypertrophic chondrocyte zone expansion seen in hypophosphatemic mice (Miedlich et al., 2010), we next sought to determine if MAPK signaling was decreased in chondrocytes isolated from *Alpl*^−/−^ mice. Consistent with previously published results, MAPK phosphorylation was induced by treatment of rib primary chondrocytes with inorganic phosphate (Figure 8). Notably, both basal and Pi induced levels of phosphorylated MAPK protein were reduced in cells isolated from *Alpl*^−/−^ mice when compared to cells isolated from *Alpl*^+/+^ mice. To determine if MAPK signaling was diminished in cranial base growth plate tissues of *Alpl*^−/−^ mice prior to the onset of obvious chondrocyte abnormalities, we next used immunohistochemistry to quantify levels of total and activated MAPK protein in ISS and SOS cranial base tissues of 5 day-old mice. Results showed that while total MAPK levels were similar in the synchondroses of *Alpl*^−/−^ and *Alpl*^+/+^ mice, phospho-MAPK levels were significantly lower in the synchondroses of *Alpl*^−/−^ mice, as compared to *Alpl*^+/+^ mice (Figure 9). More specifically, phospho-MAPK levels appeared diminished in the perichondrium, in less differentiated chondrocytes and in hypertrophic chondrocytes of *Alpl*^−/−^ mice.

## Discussion

TNAP functions to promote bone mineralization in humans and mice (Millán and Whyte, 2016; Whyte, 2016). Consistent with this known role of TNAP, *Alpl*^−/−^ cranial base cortical bone exhibits location dependent reductions in calcium phosphate mineral content. Yet excess mineral is present within the presumed marrow space of *Alpl*^−/−^ cranial base bones by 2 weeks after birth, as shown in SEM images and as measured by energy dispersive X-ray spectroscopy. This data is consistent with reports showing that hypophosphatasia patients can develop myelophthisic anemia (Millan, 2006; Mornet, 2007). The calcium to phosphate ratios of *Alpl*^−/−^ bone tissues are similar to those of control hydroxyapatite and bone tissues of *Alpl*^+/+^ mice, indicating that the mineral within *Alpl*^−/−^ marrow is apatitic. SEM images also show evidence of calcification within the intersphenoidal and sphenoccipital synchondroses of *Alpl*^−/−^ but not *Alp*^+/+^ mice at 21 days after birth. This mineral appears within the center of the synchondroses, in less mature chondrocytes. Mineral deposition normally occurs during cranial base growth but is more typically seen near the adjacent bone margins extending into the synchondrosis (Pan et al., 2013; Vora et al., 2016). That TNAP deficiency causes cortical hypomineralization but trabecular hypermineralization, plus ectopic calcification within synchondroses is of interest. It is possible that the higher mineral deposition in *Alpl*^−/−^ trabecular bone occurs as a compensatory response to the lower mineral deposition seen in *Alpl*^−/−^ cortical bone, as has been suggested for the localized regions of long bone hypermineralization that are found in children with HPP (Girschick et al., 1999, 2007). It is also possible that TNAP deficiency causes ectopic mineral deposition by less mature cells through an as yet unknown process.

Similar to that seen in long bones, cranial base bones elongate via an endochondral process involving chondrocyte proliferation, hypertrophy, apoptosis, and replacement of cartilage with bone (Wei et al., 2016). *Alpl*^−/−^ mice exhibit hypertrophic chondrocyte zone expansion in cranial base growth plates plus diminished cranial base bone growth that is rescued upon treatment with recombinant TNAP enzyme. This data provides strong evidence that TNAP mediates the cellular process of endochondral bone development and is essential for cranial base growth. These results further support the idea that TNAP plays multiple important functions during skeletal development that are in addition to its previously established role in tissue mineralization. The findings reported here also support the idea that the overall skull shape abnormalities including midface deficiency seen in *Alpl*^−/−^ mice prior to the onset of craniosynostosis (Liu et al., 2014) result at least in part from deficient growth of the cranial base.

Cranial base growth plate expansion and increased hypertrophic zone widths of *Alpl*^−/−^ mice could result from increased proliferation and differentiation of chondrocytes, and/or diminished apoptosis or removal of hypertrophic chondrocytes. To distinguish between these possibilities, and to establish that TNAP influences chondrocytes in a cell autonomous manner, we isolated primary cells from *Alpl*^−/−^ mice and wild type littermates. For these studies, we used rib chondral cells due to the challenges of obtaining sufficient cell numbers from the cranial base. *Alpl*^−/−^ primary chondrocytes displayed diminished proliferation when compared to cells isolated from wild type mice, indicating that increased proliferation was not likely the cause of growth plate expansion seen in *Alpl*^−/−^ mice. In addition, while *Alpl*^−/−^ primary chondrocytes displayed a slightly increased tendency for proteoglycan accumulation upon differentiation in culture as assessed by alcian blue staining, a time course of mRNA expression levels did not reveal a consistent pattern of enhanced chondrocyte differentiation. Sox9 is known to stimulate Col2a1 and proteoglycan expression, while suppressing Col10a1 expression in less mature chondrocytes (Leung et al., 2011). Consistent with these known functions of Sox9, Col2a1, and aggrecan mRNA levels were significantly lower in *Alpl*^−/−^ cells as compared to *Alpl*^+/+^ cells at day 3 of differentiation. Yet, despite the lower levels of Sox9 at later differentiation time points, Col2a1 levels were higher in *Alpl*^−/−^ than *Alpl*^+/+^ cells at days 7 and 15, indicating that factors other than Sox9 may be driving the higher Col2a1 gene expression at days 7 and 15 in *Alpl*^−/−^ cells. Sox5 and Sox6 act cooperatively with Sox9 in promotion of Col2a1 expression so could mediate the higher Col2a1 gene expression seen in differentiating *Alpl*^−/−^ cells (Lefebvre et al., 1998). But Sox9, Sox5, and Sox6 are known to stimulate the differentiation of mesenchymal precursors into proliferating chondrocytes but inhibit maturation into hypertrophic chondrocytes. Given that *Alpl*^−/−^ mice exhibit an expanded hypertrophic chondrocyte zone, it therefore seems unlikely that Sox9, Sox5, or Sox6 are likely to be driving the *Alpl*^−/−^ growth plate phenotype. It is also worth noting that differentiation of rib chondral cells *in vitro* may not entirely reflect differentiation of cranial base chondrocytes *in vivo*.

We next investigated altered cell death as a potential mechanism underlying the abnormal endochondral bone development seen in *Alpl*^−/−^ mice, and found that *Alpl*^−/−^ primary hypertrophic chondrocytes exhibited a diminished tendency for apoptosis *in vitro*. Consistent with this finding, annexin V and fragmented caspase 3 expression were lower in *Alpl*^−/−^ than in *Alpl*^+/+^ cranial base synchondroses at 15 days after birth, indicating that *Alpl*^−/−^ chondrocytes have a diminished tendency to undergo apoptosis. This phenomenon could account for the expanded hypertrophic chondrocyte zone seen in *Alpl*^−/−^ growth plates. Hypertrophic chondrocyte apoptosis coincides with blood vessel invasion leading to clastic removal of cartilaginous matrix and replacement with bone that is dependent upon expression of VEGF (Gerber et al., 1999; Kronenberg, 2003), therefore we also assayed for VEGF expression. *Alpl*^−/−^ primary chondrocytes expressed lower levels of VEGF mRNA than *Alpl*^+/+^ cells at all time points of differentiation and appeared to plateau at later differentiation time points. Immunofluoresecent staining for VEGF also revealed diminished VEGF expression in cranial base synchondroses and particularly in hypertrophic chondrocytes. Together, our data suggests that TNAP is essential for the later stages of endochondral bone development including the apoptosis of hypertrophic chondrocytes and VEGF mediated recruitment of blood vessels for replacement of cartilage with bone. How TNAP mediates these effects is not yet known.

Of relevance to mechanisms that might underlie the influence of TNAP on growth plate chondrocytes, hypertrophic zone expansion with diminished endochondral bone growth is also seen in genetic and diet induced mouse models of hypophosphatemia. Vitamin D receptor null mice have expanded and disorganized hypertrophic chondrocyte zones due to diminished hypertrophic chondrocyte apoptosis that are rescued upon normalization of calcium and phosphate levels via diet (Li et al., 1997, 1998; Donohue and Demay, 2002). That this defect in hypertrophic chondrocyte apoptosis occurs due to low phosphate, as opposed to high calcium levels, was demonstrated by showing similar growth plate abnormalities in Phex mutant mice, which have low phosphate but normal calcium levels (Sabbagh et al., 2005). Subsequent studies showed that inorganic phosphate induces hypertrophic chondrocyte apoptosis dependent upon MAPK signaling (Miedlich et al., 2010). Previous studies also showed that reduction of MAPK signaling through genetic and chemical technologies impairs terminal hypertrophic chondrocyte differentiation and apoptosis (El-Hoss et al., 2014; Chen et al., 2015). *Alpl*^−/−^ mice are not hypophosphatemic but TNAP is a local generator of inorganic phosphate, therefore we next sought to determine if TNAP deficiency diminishes MAPK signaling. Immunoprecipitation experiments showed diminished basal and phosphate induced levels of activated MAPK protein in *Alpl*^−/−^ as compared to *Alpl*^+/+^ primary rib chondral cell lysate. Concordantly, immunohistochemical staining showed diminished phosphorylation of MAPK protein in the intersphenoidal and sphenoccipital synchondroses of *Alpl*^−/−^ as compared to *Alpl*^+/+^ mice. Despite similar total levels of MAPK protein, phosphorylated MAPK protein was essentially absent from round and columnar chondrocytes, and was diminished in hypertrophic chondrocytes of *Alpl*^−/−^ as compared to *Alpl*^+/+^ mice at 5 days after birth, prior to the onset of gross phenotypic abnormalities. These data are consistent with the hypothesis that growth plate chondrocyte abnormalities in *Alpl*^−/−^ mice are due to diminished MAPK signaling, likely due to diminished local levels of inorganic phosphate. *Alpl*^−/−^ mice may therefore serve as an excellent model to distinguish local vs. systemic effects of phosphate on bone and cartilage. Additional studies are required to determine if decreased local inorganic phosphate levels and MAPK signaling are essential to the growth plate chondrocyte abnormalities seen in *Alpl*^−/−^ mice.

## Author contributions

NH led the conception and design of the work, analyzed and interpreted data; drafted the manuscript, approved of the final version for publication; and agrees to be accountable for all aspect of the work related to accuracy and integrity. HN, MS contributed to the intellectual design of the work, and acquired, analyzed and interpreted data; revised the draft manuscript for intellectual content; approved of the final version to be published; and agrees to be accountable for all aspect of the work related to accuracy and integrity. JL acquired and analyzed data; revised the draft manuscript for intellectual content; approved of the final version to be published; and agrees to be accountable for all aspect of the work related to accuracy and integrity.

## Funding

This work was supported in part by Alexion Pharmaceuticals, Inc. (New Haven, CT, USA) and by NIH/NIDCR grant R01 DE025827 (to NEH).

### Conflict of interest statement

This work was supported in part by Alexion Pharmaceuticals, Inc. (New Haven, CT, USA), the licensed manufacturer of asfotase alfa/strensiq(R) (recombinant mineral-targeted TNAP). The authors have no other conflicts of interest to report.

## Supplementary material

Supplementary Figure 1 **Histologic cranial base abnormalities in *Alpl*^−/−^ mice**. 10x magnification of hematoxylin and eosin stained, decalcified, cranial base sagittal sections from *Alpl*^+/+^ **(A**) and *Alpl*^−/−^ **(B**) 20 day-0ld mice are shown, including the spheno-occipital (SOS) and inter-sphenoidal (ISS) synchondroses. Well demarcated cranial base cortical bone surrounding marrow and trabecular bone is present in the *Alpl*^+/+^ mouse. In contrast, the *Alpl*^−/−^ cranial base bones lack distinct cortex, trabeculae and marrow. Additionally, both the SOS and ISS appear grossly abnormal in the *Alpl*^−/−^ cranial base, as compared to that seen in the *Alpl*^+/+^ cranial base. 40x magnification of *Alpl*^+/+^ SOS **(C**), *Alpl*^+/+^ ISS **(D**), *Alpl*^−/−^ SOS **(E)** and *Alpl*^−/−^ ISS **(F)** are shown. Note increased width of hypertrophic zones in both the SOS and ISS of *Alpl*^−/−^ mice.
