# Supplementary figures and images for: Tissue Nonspecific Alkaline Phosphatase (TNAP) Regulates Cranial Base Growth and Synchondrosis Maturation
Source: Front Physiol. 2017 Mar 21;8:161. doi: 10.3389/fphys.2017.00161 (PMC5359511; doi:10.3389/fphys.2017.00161)

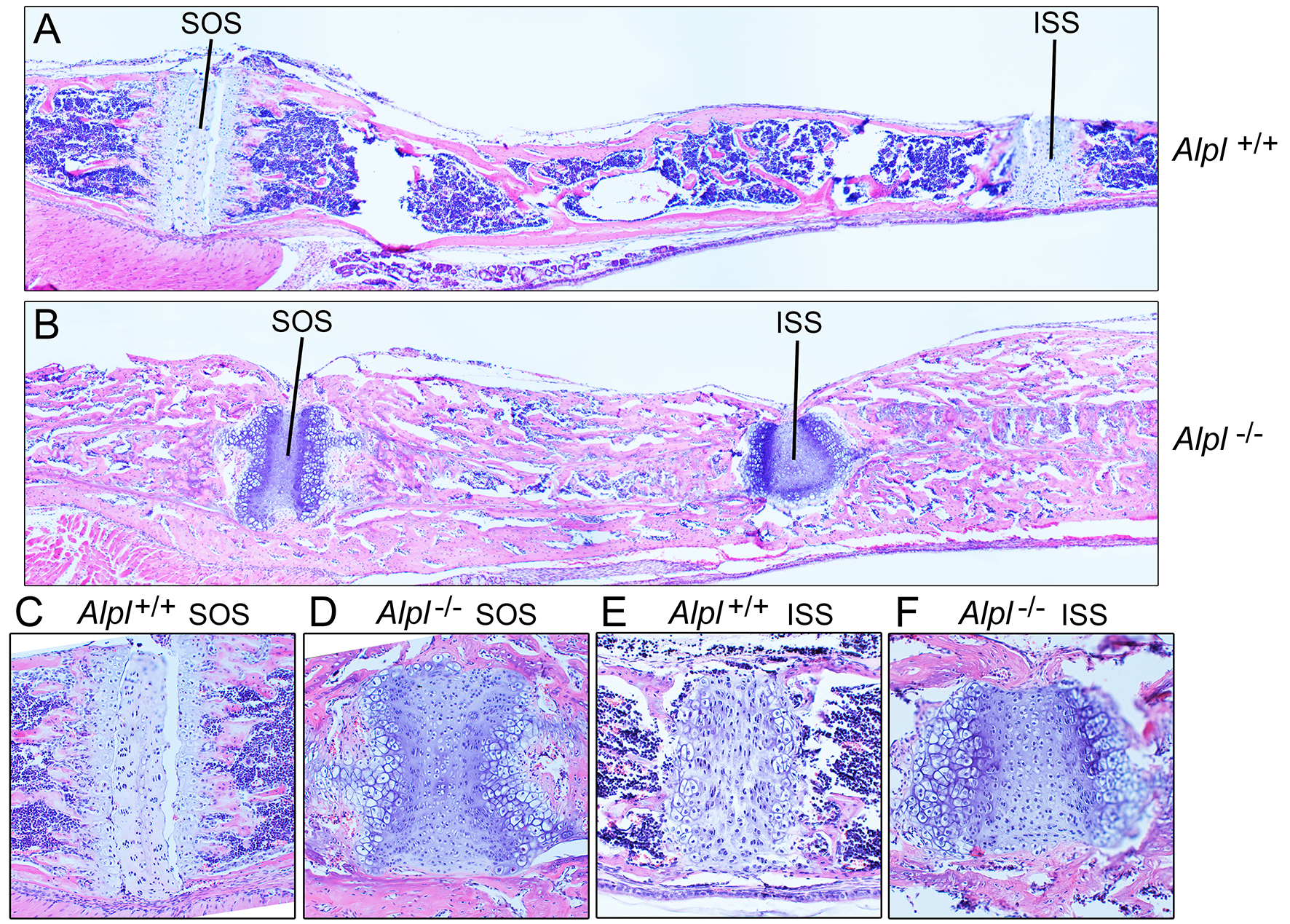

Supplement: Supplementary Figure 1 — Histologic cranial base abnormalities in Alpl−/− mice. 10x magnification of hematoxylin and eosin stained, decalcified, cranial base sagittal sections from Alpl+/+ (A) and Alpl−/− (B) 20 day-0ld mice are shown, including the spheno-occipital (SOS) and inter-sphenoidal (ISS) synchondroses. Well demarcated cranial base cortical bone surrounding marrow and trabecular bone is present in the Alpl+/+ mouse. In contrast, the Alpl−/− cranial base bones lack distinct cortex, trabeculae and marrow. Additionally, both the SOS and ISS appear grossly abnormal in the Alpl−/− cranial base, as compared to that seen in the Alpl+/+ cranial base. 40x magnification of Alpl+/+ SOS (C), Alpl+/+ ISS (D), Alpl−/− SOS (E) and Alpl−/− ISS (F) are shown. Note increased width of hypertrophic zones in both the SOS and ISS of Alpl−/− mice. [file Image1.TIF]
